# Analysis of employee diligence and mining of behavioral patterns based on portrait portrayal

**DOI:** 10.1038/s41598-024-62239-0

**Published:** 2024-05-24

**Authors:** Chiyin Wang, Yiming Liu

**Affiliations:** 1 School of Business and Economics, Universiti Putra Malaysia, 43400 Kuala Lumpur, Selangor Malaysia; 2 School of Business Management, Universiti Utara Malaysia, 06050 Sintok, Kedah Malaysia

**Keywords:** Portrait portrayal, Data mining, Diligence analysis, Abnormal behavior identification, Behavior patterns, Computer science, Information technology

## Abstract

With the deepening of enterprise digital construction, the portrait portrayal based on employee behaviors has gradually become a research focus. Currently, the employee's portrait portrayal mostly has the problems of simple means, low efficiency, limited solving ability, etc., making the results more one-sided. Therefore, a data mining-based employee portrait portrayal model is proposed. The content of employee portrait portrayal is deeply analyzed, and the overall framework of the model is designed. A diligence analysis model (DAM) based on improved GAN is constructed, and the diligence evaluation of employees is clarified to realize the diligence evaluation. The results of diligence analysis of DAM have high accuracy (80.39%) and outperform SA (70.24%), K-means (51.79%) and GAN (67.25%). The Kappa coefficient of DAM reaches 0.7384, which is highly consistent and higher than SA (0.6075), K-means (0.3711) and GAN (0.5661). The Local Outlier Factor (LOF) and Isolation Forest (IF) are used to detect abnormal behaviors on the employees, and mine the abnormal behavior patterns on different granularity time. The LSTM model (Att-LSTM) based on the attention mechanism is used to complete the prediction of employees' software usage behaviors, and analyze and summarize the characteristics of employee's behaviors from multiple perspectives. Att-LSTM predicts the best with an RMSE of 0.82983, which is better than LSTM (0.90833) and SA (0.97767); AM-LSTM has a MAPE of 0.80323, which is better than LSTM (0.86233) and SA (0.92223). The results show that the data mining-based employee portrait portrayal method can better solve the problem of enterprise employees' digital construction, and provide a new way of thinking for the construction of enterprise-level employees' digital portrait model and the analysis of employee behavior.

## Introduction

The development of new energy vehicles provides more employment opportunities for the society and has a significant role in promoting the reduction of carbon emissions. The new energy vehicle field of research and development, production, sales and other employees are in increasing demand, high level of professional staff has become an important force to promote the development of new energy vehicle companies. However, the management of employees in new energy automobile companies still adopts a relatively backward management mode, which cannot be improved with the development of new energy vehicles and the progress of the times, especially the career management of employees. Employees in production positions have low motivation and high mobility, employees in technical positions are not given enough attention by the enterprise, and their scientific research and innovation ability has not been given full play, while there are more administrative managers who are not proficient in the research and development and production of new energy vehicles. Therefore, in the process of promoting digitalization in enterprises, employee information is particularly important. A perfect employee portrait model can provide important data support for enterprise decision-making.

To efficiently complete the enterprise's supervision of employees, improve the management efficiency, enterprises need to conduct a comprehensive and integrated analysis of employees to accurately and comprehensively portray the characteristics of employee portraits^1,2^. Same as the portrait of employees in the traditional sense, nowadays the enterprise employees have a good educational background, can complete the work of technological innovation and knowledge creation through mental labor, with high comprehensive quality and strong work autonomy^3^. However, in the era of big data, new characteristics of enterprise employees have emerged. The most significant is the high-frequency active departure rate and strong job instability, which is highlighted by the strong mobility and frequent job-hopping during the career cycle. Especially in the new energy automobile industry, the phenomenon of talent mobility is particularly prominent. The high-frequency mobility of employees not only affects the decision-making of both supply and demand in the talent market, but also challenges traditional career management and employment concepts, as well as individual and organizational knowledge management.

In the big data environment, the data that can reflect the characteristics of employees is huge and complex. Therefore, the following problems inevitably exist in the process of model construction: the research related to employee portrait portrayal is not comprehensive enough; the efficiency of model analysis is low^4^. Taking enterprises as an example, most of the current research on portrait characterization is based on a single data source and fails to conduct comprehensive portrait characterization of employees from multiple perspectives, so there are problems such as inaccurate and incomplete portrait characterization^5,6^. The underlying reason for this is:

1. Huge volume of data and complex structure, such as operation logs, system data, browsing information, email data, etc.. On the one hand, the huge data system determines that enterprises can not use traditional data analysis to realize the fine-grained depiction of employee portrait characteristics^7^. On the other hand, the integration of multi-source heterogeneous data is inefficient and difficult to analyze.
2. The analysis mode of employee behavior is relatively one-sided. Existing behavioral analysis modes are mostly based on browsing data and software interaction data, and the statistical analysis process is relatively rough and mostly relies on manual analysis^8^. Therefore, the analysis of multi-source heterogeneous data cannot accurately portray employee portrait characteristics.

Therefore, to address the above problems, this paper takes the employees of a new energy automobile enterprise as the research target, and carries out the research on the employee portrait portrayal method based on data mining, focusing on the analysis of employee diligence, identification of abnormal behavior and mining of temporal behavioral patterns. The contents, standards and dimensions of employee portraits are clarified, and the general framework of employee portraits and behavioral analysis is designed. Based on data mining, combined with statistical analysis and deep learning, the Generating Adversarial Networks (GAN) model is improved to evaluate employee diligence. Isolation Forest (IF) combined with Random Forests (RF) and Local Outlier Factor (LOF) are used to analyze the abnormal behavioral characteristics of employees at different times, and to discover temporal behavioral patterns. The Long Short-Term Memory (LSTM) model based on attention mechanism was further used to predict the employee behavior and verify the model effect.

This study is based on solving the practical problems of employee portrait characterization and behavioral analysis, combining the behavioral analysis theory with data mining and deep learning to meet the rigid needs of enterprises' big data work on employees, and achieve the purpose of employee portrait characterization and behavioral analysis. This study proves the feasibility of the theoretical method, and the proposed employee portrait portrayal method and behavioral analysis method improves the accuracy of employee portrait portrayal, fully exploits the potential value of multi-source heterogeneous data, and provides theoretical basis and practical significance in the promotion of the enterprise big data construction and the behavioral analysis oriented to multi-source heterogeneous data.

## Employee portrait characterization modeling

### Content and standards of portrait characterization

#### Portrait portrayal content

Employee diligence has certain representation in employee portrait portrayal^9–11^, including job classification, work rigor, management ability, design ability and other representation, so it is necessary to use behavioral characteristics to realize employee diligence analysis. Behavioral information reflects the dynamic attributes of employees, so according to the employee behavior to mine behavioral laws and realize their behavioral prediction.

The set of employees of an enterprise is denoted as $Person = \left\{ {p_{1} ,p_{2} ,p_{3} \ldots } \right\}$. An employee generates *n* kinds of behavioral data in the enterprise activities, and the *i-*th kind of behavioral data of employee *P* is denoted by $A_{i}^{p}$. Then the set of all behavioral data of *P* can be denoted as:

$$ Action^{P} = \left\{ {A_{i}^{P} |1 \le i \le n,P \in Person} \right\} $$

#### Behavioral evaluation standards

##### Diligence

Enterprises evaluate whether an employee is diligent and how diligent he is, mainly examining his usual work attitude, conscientiousness, learning ability and planning ability. Therefore, the evaluation of diligence is mainly considered from three aspects: learning (diligent in learning), planning (diligent in planning) and working (diligent in working)^12^.

*Diligent in learning*: Mainly refers to the ability of employees to learn frequently to improve their ability and work efficiency, usually manifested in book browsing, website browsing, and the use of professional software. Studious employees have a higher frequency of book browsing, longer browsing time, and longer time and frequency of use of professional software.

*Diligent in planning*: To examine whether they plan in advance, and whether they are capable of executing the plan and the degree of execution, usually manifested in the usual planning, such as the number of planning, the finish rate of planning, the timeliness of planning, and so on. Diligent employees generally have strong planning skills and are able to make reasonable plans and complete them in a timely manner.

*Diligence in work*: To examine whether the working time of the employees is reasonable, how efficient they are, and how the quality of the tasks is accomplished, usually manifested in attendance, task completion, website browsing, use of professional software, use of public storage, and so on. In general, relatively hard-working employees have higher attendance, on-time, overtime, frequency of professional website browsing and use.

*For "diligent in learning"*: Book browsing time is *book_t*, browsing frequency is *book_f*; browsing time of learning website is *web_s_t*, browsing frequency is *web_s_f*; browsing time of work website is *web_w_t* and browsing frequency is *web_w_f*; time in using software is *software_t* and frequency is *software_f*. Assuming that each factor has its corresponding importance indicator $w_{i}^{s}$, the "diligent in learning" can be expressed as follows:

$$ \begin{gathered} Learning = \left[ {\begin{array}{*{20}l} {w_{1}^{s} } \hfill & {w_{2}^{s} } \hfill & \cdots \hfill & {w_{n}^{s} } \hfill \\ \end{array} } \right]\left[ {\begin{array}{*{20}l} {book_{ - } t} \hfill & {book_{ - } f} \hfill & \cdots \hfill & { \, software_{ - } f} \hfill \\ \end{array} } \right]^{T} \\ = w_{1}^{s} book_{ - } t + w_{2}^{s} book_{ - } f + \cdots + w_{n}^{s} software_{ - } f \\ \end{gathered} $$

*For "diligent in planning"* The number of planning is *planning_n*, the finish rate of planning is *planning_f*, and the timeliness of planning is *planning_t*. Assuming that each factor has its own corresponding importance indicator $w_{i}^{p}$, the "diligent in planning" can be expressed as follows:

$$ \begin{gathered} Planning = \left[ {\begin{array}{*{20}l} {w_{1}^{p} } \hfill & {w_{2}^{p} } \hfill & \cdots \hfill & {w_{m}^{p} } \hfill \\ \end{array} } \right][planning\_n \, planning\_f \, \cdots planning\_t]^{T} \\ = w_{1}^{p} planning\_n + w_{2}^{p} planning\_f + \cdots + w_{m}^{p} planning\_t \\ \end{gathered} $$

*For "diligent in working"* Attendance frequency is *attendance_f*, on-time frequency is *on Time_f*, and overtime frequency is *overtime_f*; browsing frequency of professional network is *professional_network_f*, the usage frequency of professional network is *professional_software_f*^13,14^. Assuming that each factor has its corresponding importance indicator $w_{i}^{w}$, the "diligent in working" can be expressed as follows:

$$ \begin{gathered} Working = \left[ {\begin{array}{*{20}l} {w_{1}^{w} } \hfill & {w_{2}^{w} } \hfill & {w_{3}^{w} } \hfill & \cdots \hfill \\ \end{array} } \right]\left[ {\begin{array}{*{20}l} {attendance\_f} \hfill & {on \, Time\_t} \hfill & {overtime\_f} \hfill & \cdots \hfill \\ \end{array} } \right]^{T} \\ = w_{1}^{w} attendance\_f + w_{2}^{w} onTime\_f + w_{3}^{w} overtime\_f + \cdots \\ \end{gathered} $$

The employee's diligence score *Score* can eventually be expressed as:

$$ Score = Learning + Planning + Working $$

Assuming that the scoring quasi-line is *Q*_1_, *Q*_2_, *Q*_3_, *Q*_4_, *Q*_5_, and *Q*_6_, then the diligence level can be divided as:

$$ Diligent - type = \left\{ {\begin{array}{*{20}c} {Extremely \, Diligent} & {Q_{1} < \, Score \, \le Q_{2} } \\ {General \, Diligent} & {Q_{2} < \, Score \, \le Q_{3} } \\ {Ordinary} & {Q_{3} < \, Score \, \le Q_{4} } \\ {Slack} & {Q_{4} < \, Score \, \le Q_{5} } \\ {Extremely \, Slack} & {Q_{5} < \, Score \, \le Q_{6} } \\ \end{array} } \right. $$

##### Behavior

*Detection of abnormal behaviors*: According to the feature weights, *N* behavioral features are selected and the Local Outlier Factor (LOF) algorithm is applied to calculate their different detection thresholds *E* for different functions. If an employee's behavior_ *Action*^*P*^ exceeds the detection threshold *E*, it is regarded as abnormal behavior:

$$ \_Action^{P} > E $$

Employees whose number of abnormal behaviors exceeds a certain threshold are considered as abnormal employees, and the set of abnormal behaviors of employee *P* is denoted as:

$$ Action^{P} = \left\{ {A_{1} ,A_{2} , \ldots A_{n} } \right\} $$

If the number of abnormal behaviors *n* > *k*, the employees are determined to be abnormal employees.

The screened employees with a large number of abnormal behaviors are mined from the perspective of time at different granularities^15^. Adopting the Isolation Forest (IF), the abnormal values on each time period is calculated, and the time period exceeding the abnormal threshold is regarded as abnormal time, so as to determine the time when the abnormal behavior occurs.

*Prediction of temporal behaviors*: The Long Short-Term Memory (LSTM) model based on attention mechanism is used to analyze the temporal correlation of the historical behavior of software usage and make predictions for new behaviors^16^. Assuming that the real behavior of an employee in a certain time period is *base_ action*' and the prediction of the behavior is *prediction_action*_*i*_ using the prediction model *F*^*i*^, the model can be expressed as:

$$ E_{i} = f\left( {base\_action^{\prime}, \, prediction\_action_{i} } \right) \le \varepsilon $$

$$ prediction\_action_{i} = F^{i} (history\_action) $$

where *history_action* denotes historical behavioral data; *f* denotes evaluation metrics; *E*_*i*_ denotes the evaluation result of model *F*_*i*_; *ε* is a threshold line for evaluating the model. The model needs to satisfy the evaluation result *E*_*i*_ does not exceed the threshold line, i.e. $E_{i} \le \varepsilon$.

### Process of portrait characterization

**Figure 1:**
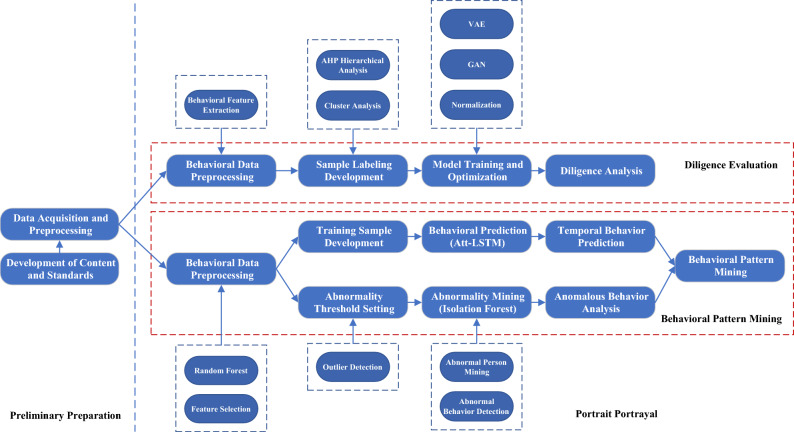
Process of employee portrait portrayal.

Combined with the actual workflow of a new energy automobile enterprise, the design of the employee portrait portrayal process is shown in Fig. 1. The process of employee portrait portrayal mainly includes five parts: portrait portrayal content and standard, data processing and feature extraction, diligence analysis, abnormal behavioral analysis, and temporal behavioral prediction. The pre-preparation stage includes the content and standards of employee portrait portrayal and data processing. Diligence evaluation and behavioral mining are the two parallel dimensions of portrait carving, in which the temporal behavioral law includes two parts: abnormal behavioral detection and temporal behavioral prediction.

## Employee diligence analysis

### Diligence criteria

Enterprises evaluate whether employees are diligent or not usually based on the diligence evaluation index to assess employees. Therefore, this paper adopts classical Statistical Analysis (SA), Random Forest (RF), Isolation Forest (IF) and Analytic Hierarchy Process (AHP) with human subjective evaluation factors to comprehensively evaluate the employee diligence and formulate the diligence criteria.

#### Diligence evaluation index

Diligence evaluation index refers to the evaluation dimensions of employee diligence, i.e., from which angles should employee diligence be evaluated (on-time frequency, overtime frequency, etc.)^17^. Using employee behavior data to evaluate the diligence *Q*_*P*_ of employee *P*, there are *k* indicators to form the evaluation system of the employee, i.e., to evaluate the employee diligence from *k* dimensions, then the indicator set *Tar* is expressed as:

$$ Tar = \left\{ {T_{j} |1 \le j \le k} \right\} $$

where *T*_*j*_ denotes the *j*-th indicator in the indicator set *Tar*, which contains behavioral data as $A^{j} = \left\{ {a_{1}^{j} ,a_{2}^{j} ,a_{3}^{j} , \ldots } \right\}$. Therefore, it can be set that the behavioral data is the set of data contained in *k* indicators:

$$ \_Action = \left\{ {A_{j} |1 \le j \le k,A_{j} \in Action,\_Action \subseteq Action} \right\} $$

Doing feature extraction on the employee behavior data _*Action*, the feature vector with *m* features can be denoted as $$A = \left[ {\begin{array}{*{20}l} {a_{1} } \hfill & {a_{2} } \hfill & \ldots \hfill & {a_{m} } \hfill \\ \end{array} } \right]^{T}$$, and $a_{i} (1 \le i \le m)$ denotes the *i-*th behavioral feature. *N* sets of time intervals spanning *t* are formed into a time series $$A = \left[ {\begin{array}{*{20}l} {t_{1} } \hfill & {t_{2} } \hfill & \ldots \hfill & {a_{n} } \hfill \\ \end{array} } \right]$$ to construct a matrix of behavioral characteristics with time series:

$$ A = \left[ {\begin{array}{*{20}c} {a_{1} } \\ {a_{2} } \\ \ldots \\ {a_{m} } \\ \end{array} } \right] \times \left[ {\begin{array}{*{20}c} {t_{1} } \\ {t_{2} } \\ \ldots \\ {t_{n} } \\ \end{array} } \right]^{T} = \left[ {\begin{array}{*{20}c} {a_{11} } & {a_{12} } & \ldots & {a_{1n} } \\ {a_{21} } & {a_{22} } & \ldots & {a_{2n} } \\ \ldots & \ldots & \ldots & \ldots \\ {a_{n1} } & {a_{n2} } & \ldots & {a_{mn} } \\ \end{array} } \right] $$

#### Weight calculation

Employee diligence is embodied in the behavioral data, which has a certain degree of variability, i.e., different behavioral data have different contribution values to diligence^18^. Drawing on the RF, the introduction of weights *w* to make the results of diligence calculation more accurate and reasonable as much as possible.

Sampling with put-back from the sample produces the training set *D*_*t*_, training is performed to produce *G*_*t*_, and multiple *G*_*t*_ are combined to get the final processing model *G*. Theoretically, there will be a portion of the samples that are not selected to train the model *G* (number 1/*e*), enough to perform the validation of the model. Out-of-bag samples are utilized for model evaluation and tuning:

$$ E_{oob} (G) = \frac{1}{N}\sum\limits_{i = 1}^{N} {err} \left( {y_{n} ,G_{n}^{ - } \left( {x_{n} } \right)} \right) $$

The importance of various behavioral features is calculated using RF:

$$ im(i) = E_{oob} (G) - E_{oob}^{P} (G) $$

$E_{oob} (G)$ denotes the error calculated using out-of-bag samples, and $E_{oob}^{P} (G)$ denotes the superposition of a feature from one of the out-of-bag samples with random noise.

The importance of each feature is calculated one by one in this way. The dataset containing *N* samples is defined as $D_{N} = \left\{ {x_{1} ,x_{2} , \ldots ,x_{N} } \right\}$. The application of RF allows to obtain the evaluated value of each feature to be used as the weight *w* of the feature value.

#### Indicator calculation

After feature extraction and weights calculation, the behavioral data need to be normalized in order to eliminate the influence of the data magnitude. Minimum–maximum normalization algorithm (Min–Max) is used to do the normalization of the data:

$$ X_{normalize \, } = \frac{{X - X_{\min } }}{{X_{\max } - X_{\min } }} $$

where *X* represents the feature data that need to be normalized, *X*_*normalize*_ represents the value of *X* after normalization, and *X*_*min*_ and *X*_*max*_ are the minimum and maximum values of the feature data, respectively^19,20^.

Due to the large number of forms of behavioral data, the formula is modified to facilitate the calculation:

$$ X_{normalize \, } = a + \frac{{(b - a)^{*} \left( {X - X_{\min } } \right)}}{{X_{\max } - X_{\min } }} $$

where *a* and *b* represent the range of normalizing the data to [*a*, *b*].

After data normalization, the joint matrix of weights and eigenvalues needs to be constructed, then the diligence eigenmatrix can be expressed as:

$$ A = \left[ {\begin{array}{*{20}c} {w_{1} a_{1} } \\ {w_{2} a_{2} } \\ \ldots \\ {w_{m} a_{m} } \\ \end{array} } \right] \times \left[ {\begin{array}{*{20}c} {t_{1} } \\ {t_{2} } \\ \ldots \\ {t_{n} } \\ \end{array} } \right]^{T} = \left[ {\begin{array}{*{20}c} {w_{1} a_{11} } & {w_{1} a_{12} } & \ldots & {w_{1} a_{1n} } \\ {w_{2} a_{21} } & {w_{2} a_{22} } & \ldots & {w_{2} a_{2n} } \\ \ldots & \ldots & \ldots & \ldots \\ {w_{m} a_{n1} } & {w_{m} a_{n2} } & \ldots & {w_{m} a_{mn} } \\ \end{array} } \right] $$

For employee *P*, further feature extraction is done using RMS Amplitude, and diligence at time period *T* is:

$$ Q_{ - } R = \sqrt {\frac{1}{T}\sum\limits_{i = 1}^{T} {q_{t}^{2} } } $$

$$ q_{t} = w_{t} a_{t1} + w_{t} a_{t2} + \cdots + w_{t} a_{tn} $$

where, *q*_*t*_ denotes the *t*-th diligence value, $t \in T$, *Q_R* denotes the final diligence result and *m* denotes the number of behavioral features.

#### Diligence classification

For the result of diligence calculation *Q_R*, the set $Q = \left\{ {Q_{i} |1 \le i \le 6} \right\}$ is defined to delineate the five grade intervals of diligence, all participants are sorted according to the diligence value from the largest to the smallest:

$$ x_{i} = (i - 1)(m + 1)/5 $$

$$ Q_{i} = F\left( {\left\lfloor {x_{i} } \right\rfloor } \right) \cdot \left( {1 - x_{i} \% 1} \right) + F\left( {\left\lceil {x_{i} } \right\rceil } \right) \cdot \left( {x_{i} \% 1} \right) $$

where *x*_*i*_ is the position, $x_{i} \in \{ 20\% ,40\% ,60\% ,80\% \}$, $1 \le i \le 5$, *i* is an integer; *m* is the total number of participants; $\left\lfloor {x_{i} } \right\rfloor$ on behalf of *x*_*i*_ downward rounding, $\left\lceil {x_{i} } \right\rceil$ on behalf of *x*_*i*_ upward rounding, *x*_*i*_%1 on behalf of *x*_*i*_ to 1 to take the remainder; *F*(*x*) that take the diligence value of the employee at position *x*.

Combined with the actual cognition of enterprise employees, diligence can be divided into "Diligent", "Ordinary", "Slack".

"Diligent" refers to employees who are active in work, and is subdivided into "Extremely Diligent" and "General Diligent". "Ordinary" employees are ordinary in work, and the evaluation index tends to be the average value. "Slack" refers to employees with poor performance in work, subdivided into "Slack" and "Extremely Slack". Therefore, the classification of diligence is expressed as follows:

$$ Diligent - type = \left\{ {\begin{array}{*{20}c} {Extremely \, Diligent} & {Q_{1} < \, Score \, \le Q_{2} } \\ {General \, Diligent} & {Q_{2} < \, Score \, \le Q_{3} } \\ {Ordinary} & {Q_{3} < \, Score \, \le Q_{4} } \\ {Slack} & {Q_{4} < \, Score \, \le Q_{5} } \\ {Extremely \, Slack} & {Q_{5} < \, Score \, \le Q_{6} } \\ \end{array} } \right. $$

### Data processing

To ensure that the evaluation of diligence has a certain objectivity and basis, it must be combined with on-site research, i.e. human evaluation. Therefore, it is necessary to use AHP analysis: constructing judgment matrix, hierarchical sorting and consistency test, weight matrix construction, and score calculation.

### Judgment matrix

The influencing factors are defined, such as attendance frequency, on-time frequency, etc.; two by two comparison of the influencing factors, to determine the proportion of each influencing factor; according to the proportion of the construction of the judgment matrix. With *n* influence factors of diligence, denoted as $F = \left\{ {f_{i} |1 \le i \le n} \right\}$, the judgment matrix is obtained by two-by-two comparison between influence factors, where *J*_*lm*_ denotes the result of comparison between *f*_*l*_ and *f*_*m*_:

$$ Judgment = \left[ {\begin{array}{*{20}c} {J_{11} } & {J_{12} } & \ldots & {J_{1n} } \\ {J_{21} } & {J_{22} } & \ldots & {J_{2n} } \\ \ldots & \ldots & \ldots & \ldots \\ {J_{n1} } & {J_{n2} } & \ldots & {J_{nn} } \\ \end{array} } \right] $$

#### Hierarchical sorting and consistency test

Hierarchical sorting of the judgment matrix and calculation of the weight coefficients using the ensemble product method; consistency test is done to eliminate contradictory values in the judgment matrix. The judgment matrix is normalized by columns:

$$ \overline{J}_{lm} = \frac{{J_{lm} }}{{\sum\limits_{i = 1}^{n} {J_{im} } }}(l,m = 1,2, \ldots ,n) $$

After the normalization, the row vectors of the judgment matrix are derived:

$$ \overline{w}_{l} = \sum\limits_{m = 1}^{n} {\overline{J}_{lm} } (l = 1,2, \ldots ,n) $$

Normalized processing vectors:

$$ w_{l} = \frac{{\overline{w}_{l} }}{{\sum\limits_{m = 1}^{n} {\overline{w}_{m} } }}(l = 1,2, \ldots ,n) $$

#### Constructing the weight matrix and calculating the diligence score

During the research process, the indicators of employee diligence are scored according to the scale 1–10, and the weight vector *w*_*l*_ is obtained to construct the weight matrix. The final diligence score of Employee *P* is equal to the cumulative sum of the product of its scores and weights, as shown in the following equation. *S*_*p*_ is the final diligence score of employee *P*, *P*_*l*_ is the *l-*th index score of employee *P*, and *w*_*l*_ is the *l-*th weight.

$$ S_{p} = \sum\limits_{l}^{n} {w_{l} } \cdot P_{l} $$

#### Diligence classification

According to "3.1.4", the data normalization range is set to [0, 100], and the final result of diligence is normalized to facilitate the score comparison. By assigning scores, *Q*_1_ = 0, *Q*_2_ = 66.9, *Q*_3_ = 72.3, *Q*_4_ = 80.6, *Q*_5_ = 83.5, and *Q*_6_ = 100 were obtained. Employees' scores in different intervals corresponded to different types of diligence. After feature normalization, training labels are made according to the score of employee diligence, as shown in Table 1:

**Table 1:** Examples of training labels (part).

No.	Attendance frequency	On-time frequency	Overtime frequency	Time of browsing website during working time	Time of browsing website during spare time	Time in using professional software	Diligence values	…	Types of diligence
175,384	85.3%	81.9%	12.5%	4.5 h	2.3 h	5.3 h	89.4	…	Extremely Diligent
175,392	73.7%	56.8%	21.4%	2.7 h	2.1 h	4.4 h	84.6	…	Extremely Diligent
175,547	75.1%	76.4%	24.5%	2.1 h	0 h	2.5 h	78.5	…	Ordinary
175,671	56.8%	33.9%	1.05%	3.7 h	1.4 h	4.6 h	82.1	…	Generally diligent
…	…	…	…	…	…	…	…	…	…
176,942	85.7%	75.7%	11.4%	3.2 h	0.8 h	4.8 h	64.3	…	Extremely Slack

### Model design

**Figure 2:**
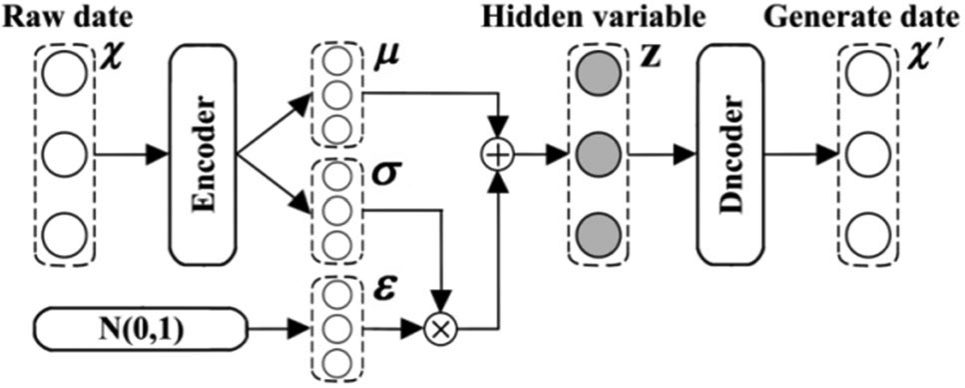
Structure of VAE.

Drawing on the traditional GAN, CGAN and DCGAN structures and incorporating the Variational Auto-Encoder (VAE), a Diligence Analysis model (DAM) is proposed. A hidden variable *z* is constructed such that it satisfies the distribution from *z* to the target data *x*', i.e., $x^{\prime } = g(z)$, so that the target data *x*' is as close as possible to the distribution of the real data *x*. For *K* samples, the Gaussian distribution for each sample is assumed to be $N\left( {\mu_{k} ,\sigma_{k}^{2} } \right)$. The VAE makes each Gaussian distribution converge to the standard Gaussian distribution *N*(0,1) as much as possible, as shown in Fig. 2.

The KL scatter is used to calculate the loss. Assume that *z* obeys a standard Gaussian distribution and the prior distribution $P(x|z)$ is Gaussian, i.e., $x|z\sim N(\mu (z),\sigma (z))$. Where $\mu (z)$ and $\sigma (z)$ are two functions that are the mean and variance of the Gaussian distribution corresponding to *z*. Then *P*(*x*) is the cumulative of all Gaussian distributions over the integration domain. The original data *x* obeys a probability distribution:

$$ P(x) = \int_{z} P (x)P(x|z)dz $$

Solving the problem is really about *µ* and *σ*. The initial goal is to solve for *P*(*x*) and to have *P*(*x*) as large as possible, and these two functions are equivalent to solving for the maximum log likelihood about *x*:

$$ L = \sum\limits_{x} {\log } P(x) $$

$$ \log P(x) = \int_{z} q (z|x)\log \left( {\frac{P(x|z)P(\xi )}{{q(z|x)}}} \right)dz + \int_{z} q (z|x)\log \left( {\frac{q(z|x)}{{P(z|x)}}} \right)dt $$

By means of the KL dispersion measure of the proximity of $q(z|x)$ to $P(x|z)$, the above equation can be transformed into:

$$ \log P(x) = L_{b} + KL(q(z|x)|P(x|z)) $$

To maximize *L*_*b*_ and minimize KL scatter, *L*_*b*_ is represented by the transformation as:

$$ L_{b} = - KL(q(z|x)|P(z)) + E_{q(\xi |x)} [\log (P(x|z))] $$

When sampling *z* from a Gaussian distribution $N\left( {\mu_{k} ,\sigma_{k}^{2} } \right)$, it is actually equivalent to sampling *ε* from *N*(0,1) and then calculating $z = \mu + \varepsilon \times \sigma$.

**Figure 3:**
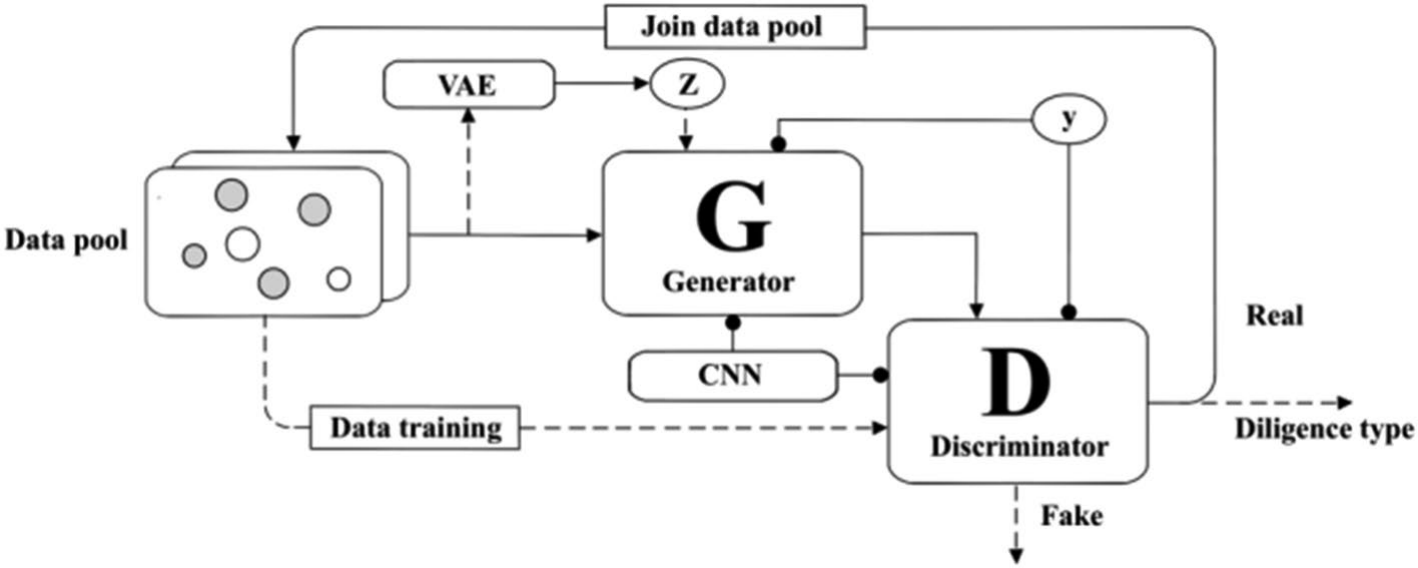
Structure of DAM.

Traditional GAN networks have great arbitrariness in generating non-image data, and there are problems such as inaccurate data generation. For this reason, Convolutional Neural Networks (CNN) are introduced with supervised learning, drawing on DCGAN. The convolutional layer and pooling layer are formulated as:

$$ Z_{j}^{i} = f\left( {\sum\limits_{i = 1}^{N} {Z_{i}^{l - 1} } \cdot w_{ij}^{l} + b_{j}^{i} } \right) $$

$$ P_{j}^{l} = f\left( {\alpha_{j}^{l} F_{d} \left( {Z_{j}^{l} - 1} \right) + b_{j}^{l} } \right) $$

where $Z_{j}^{i}$ is the *i*-th convolutional map in the *j-*th convolutional layer; $Z_{j}^{i - 1}$ is the *i-*th convolutional map in the previous layer; $w_{ij}^{l}$ is the weight of the *j-*th convolutional kernel of the *l*-convolutional layer doing the *i-*th operation; $b_{j}^{i}$ is the bias of the *j-*th convolutional kernel of the *l*-convolutional layer. Where $P_{j}^{l}$ is the *j-*th feature map in the *l*-th pooling layer; $\alpha_{j}^{l}$ is the multiplicative bias of the feature map; $F_{d} \left( x \right)$ is the downsampling function. Then the modified DAM structure is shown in Fig. 3. The pseudocode form of the DAM algorithm is shown in Table 1A, and "/**/" indicates the annotation. (Table 1A is presented in the Appendix, as is Table 2A below.)

### Testing and analysis

The employee behavior data previously selected using AHP is used as a training dataset, and a number of features from 83 behavioral features are selected for testing and analyzed using Statistical Analysis (SA)^21^, K-Means^22^, GAN^23^, and DAM, respectively. Table 2 shows the mapping of symbolic expressions corresponding to the analysis results of the four models.

**Table 2:** Symbolic mapping.

Types	Real statistics	SA	K-means	GAN	DAM
Extremely Diligent	R_ed	T_ed	K_ed	G_ed	S_ed
General diligent	R_gd	T_gd	K_gd	G_gd	S_gd
Ordinary	R_o	T_o	K_o	G_o	S_o
Slack	R_s	T_s	K_s	G_s	S_s
Extremely slack	R_es	T_es	K_es	G_es	S_es

#### Coarse-grained accuracy analysis

**Figure 4:**
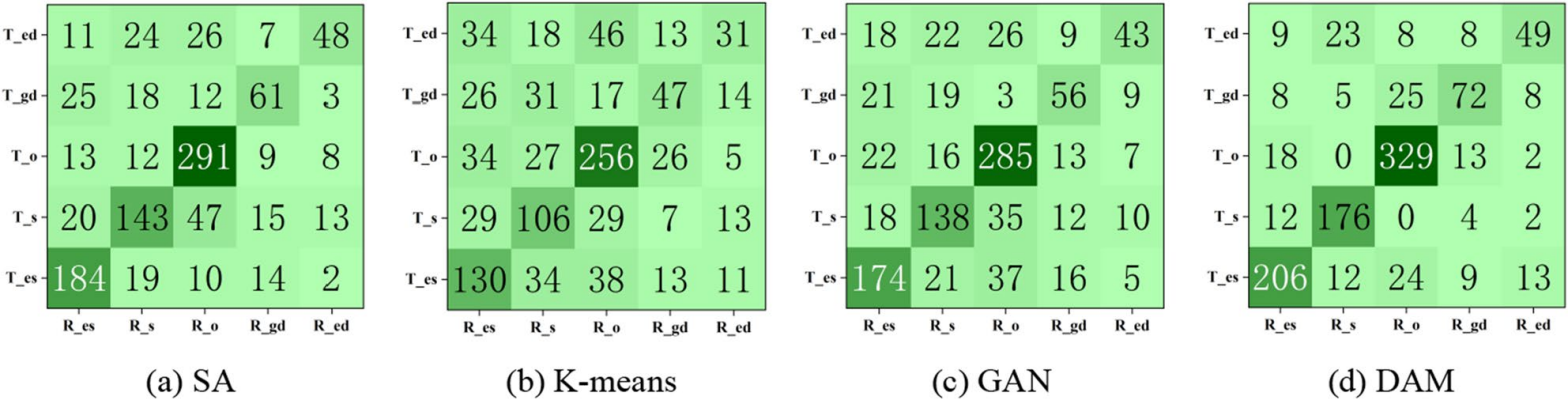
Confusion matrix of four methods. (**a**) SA, (**b**) K-means, (**c**) GAN, (**d**) DAM.

Figure 4 shows the confusion matrix of the four diligence analysis methods under the 83 behavioral features, (a), (b), (c), and (d) are the results of the confusion matrices of SA, K-Means, GAN, and DAM, respectively. The data located in the dark-colored region of the main diagonal is the number of employees correctly classified, and the data located on both sides of the main diagonal is the number of employees not correctly classified. It can be seen that the confusion matrix main diagonal data of the DAM (832) is higher than the other models (GAN: 696, K-means: 570, and SA: 727), and the actual diligence analysis results are better than the other models.

#### Fine-grained accuracy analysis

To further compare the results, the first 42 behavioral features and 83 behavioral features were selected to be evaluated using four methods for diligence evaluation, and the advantages and disadvantages of the four methods were derived using precision (P), recall (R), and accuracy (Acc) as shown in Table 3. It can be seen that the SA of diligence analysis has a certain degree of accuracy. K-Means is less accurate among the diligence analysis, and as the features increase, the accuracy of the generated data decreases instead. This is because the K-Means is an unsupervised learning, and its classification results are not directed to "diligence". The accuracy of the GAN is slightly better than K-Means, and less accurate than SA. This is because GAN has the advantage in image generation, but its performance in data generation is not good enough, and its accuracy is low, which leads to the poor classification results of diligence. The diligence analysis results of the DAM proposed in this paper have high accuracy and perform better than the other three methods.

**Table 3:** Data comparison of different models.

Methods	Diligence types		Number of features					
			42			83		
			P(%)	R(%)	Acc(%)	P(%)	R(%)	Acc(%)
SA	ed	Extremely diligent	71.43	67.19	67.44	77.31	72.73	70.24
	ge	General Diligent	60.08	66.20		62.32	68.47	
	o	Ordinary	83.64	71.50		87.39	75.39	
	s	Slack	45.52	57.55		51.26	56.41	
	es	Extremely Slack	40.68	64.86		41.38	66.59	
K-means	ed	Extremely Diligent	57.52	51.38	55.07	54.50	45.45	51.79
	ge	General Diligent	57.61	49.07		55.21	49.07	
	o	Ordinary	73.56	66.32		71.39	61.40	
	s	Slack	34.81	44.34		30.52	44.34	
	es	Extremely Slack	21.83	41.89		21.23	41.89	
GAN	ed	Extremely Diligent	63.91	58.10	59.23	68.77	68.77	67.25
	ge	General Diligent	58.65	56.48		64.79	63.89	
	o	Ordinary	80.48	69.43		83.09	73.83	
	s	Slack	33.60	39.62		51.85	52.83	
	es	Extremely Slack	24.46	45.95		36.44	58.11	
DAM	ed	Extremely Diligent	76.03	72.73	77.49	78.03	81.42	80.39
	ge	General Diligent	90.32	77.78		90.72	81.48	
	o	Ordinary	89.16	85.23		90.88	86.08	
	s	Slack	57.14	67.92		61.02	68.83	
	es	Extremely Slack	43.75	66.22		50.52	69.74	

#### Model performance and consistency test

**Figure 5:**
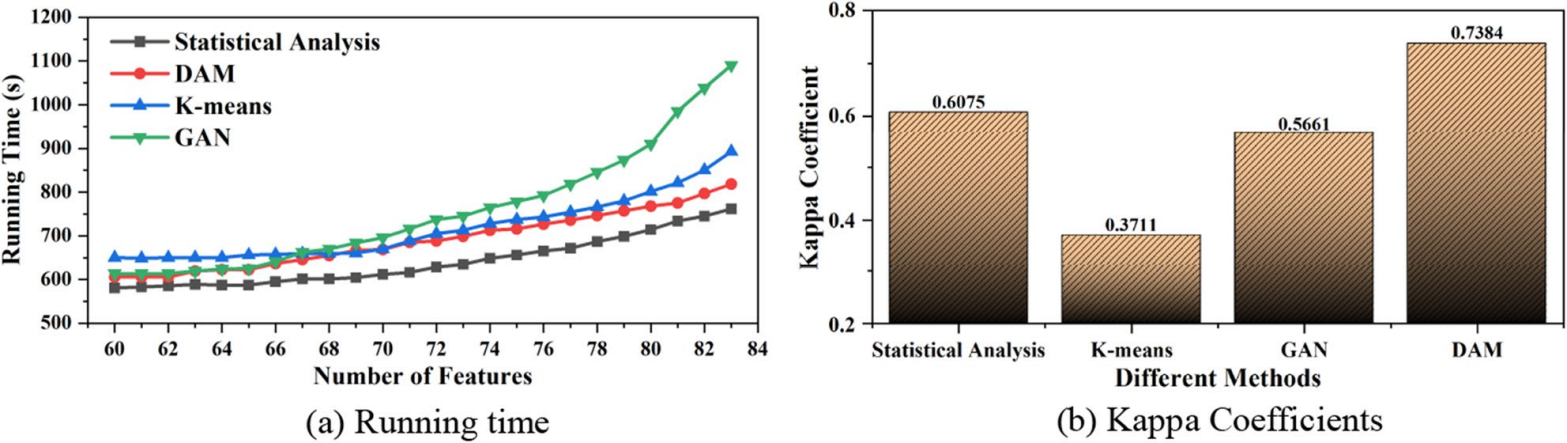
Accuracy comparison. (**a**) Running time, (**b**) Kappa Coefficients.

Figure 5 shows the efficiency and Kappa coefficient of the four methods are compared. Figure 5a shows the comparison of the running time as the feature dimensions increase. It can be seen that SA takes the least time to analyze when analyzing employee diligence, DAM is second only to the SA, K-Means takes slightly more time than GAN when the feature dimension is 20, and with the increase of feature dimension, GAN takes gradually more time than K-Means. With the increase of feature dimensions, the time consuming of four types grows slightly, but the increase of feature dimensions makes the diligence analysis results also more accurate, and the Kappa coefficient also increases. As shown in Fig. 5b, the Kappa coefficient of DAM reaches 0.7384 when the feature dimension reaches 83, which is more consistent and higher than other models. Although DAM is slightly lower than the SA in terms of running time, but there is a great increase in the accuracy of the diligence analysis results, so the comprehensive consideration, DAM is more excellent in the analysis of employee diligence, and can achieve more satisfactory results.

### Ethical statement

All methods were carried out in accordance with relevant guidelines and regulations. The research project has been supervised and approved by the ethics review committee of Universiti Putra Malaysia (number: UPM-2023012144). Informed consent was obtained from all subjects and there were no minors. Participants could not be identified in all of the data.

## Abnormal behavioral detection

### Definition of behavioral characteristics

Employee behavior characteristics can be described in terms of roles, objects, time, and types, and information of employees is its behavioral characteristics and constitutes a behavioral feature space:

$$ f_{i} = \{ r \in Role, \, b \in Behavior\_object,t \in Time, \, bt \in Behavior\_type,v \in Value\} $$

The feature space corresponding to each employee constitutes a feature vector, which together form the eigenvector space $S = \left\{ {f_{i} |i = 1,2, \ldots n} \right\}$.

*Role*: Functions and roles are categorized into three types, managers, technicians, operators. According to the function, role characteristics respectively to establish their respective feature vector, can make the description of features more objective and accurate. *Behavior_object*: refers to the collection of behaviors of employees in enterprise activities, such as access control card, network browsing, professional daily office software OA system operation. *Time*: Behavioral time, which encompasses different scales of time ranges, or time slices of different granularity sizes. Employee behavior can be analyzed from a time perspective, and can be counted by days, weeks, months, quarters, years, and other time slices of different granularity. Days can be divided into hours, minutes, seconds and other finer-grained time slices, which can be used to analyze the real-time behavior of employees; behavioral analysis in terms of days can be used to analyze the work of employees, such as overtime, etc.; in terms of weeks can be used to analyze the daily work of employees and the work of rest days. *Behavior_type*: a collection of employee behavior types, indicating a collection of behavior types of employees, such as uploading, undoing, releasing, etc. in OA interactions, or receiving and sending emails. *Value*: Behavioral indicators, such as cumulative number of values, ratios, ratios, etc., for example, the length of time and usage rate of daily office software for managers, and the ratio of professional software to daily working time for technicians^24^.

Assuming that samples are randomly drawn from the dataset *U* in a releasing manner to obtain the training set *T*. Assuming that the number of sample draws is *n*, a total of *n* training sets, denoted as $Train = \left\{ {T_{i} |i = 1,2, \ldots ,n} \right\}$, are generated. Each training set generates a training *f*_*i*_, and multiple *f*_*i*_ are combined to obtain the final model *F*. Only 1–1/ *e* proportion of the samples will be added to the training set, and the remaining data is the "out-of-bag data"^25^. The objective function is:

$$ E(F) = \frac{1}{N}\sum\limits_{i = 1}^{n} {\text{err}} \left( {y_{n} ,F_{n}^{ - } \left( {x_{n} } \right)} \right) $$

where (*x*_*n*_, *y*_*n*_) denotes samples not involved in training and $F_{n}^{ - } \left( {x_{n} } \right)$ denotes sample training. Assuming that there is a feature set of $Characters = \left\{ {c_{i} |i = 1,2, \ldots ,m} \right\}$, importance assessment is performed using RF:

$$ E(F) = \frac{1}{N}\sum\limits_{i = 1}^{n} {\text{err}} \left( {y_{n} ,F_{n}^{ - } \left( {x_{n} } \right)} \right) $$

where *E*(*F*) denotes the error computed for out-of-bag samples and *E*^*P*^(*F*) denotes the random error superimposed on a feature for an out-of-bag sample. In this way the importance of the features is evaluated and hence the features are selected based on the importance value.

Assuming that there is a dataset $U_{N} = \left\{ {\left( {x_{i} ,y_{i} } \right)|i = 1,2, \ldots ,N} \right\}$ containing *N* samples, each containing *m* features, denoted as $x_{i} = \left\{ {x_{i}^{1} ,x_{i}^{2} , \ldots ,x_{i}^{m} } \right\}$, and each sample belonging to a functional role *y*_*i*_, the strong correlation features can be selected by ranking the feature importance properties in decreasing order.

### Identification of abnormal employee behaviors

Local Outlier Factor (LOF) is a density-based sample distribution detection algorithm that determines whether a sample *x* is anomalous or not by comparing the density of each sample *x* with the density of the sample points in its neighboring region^26^. In detecting abnormal behaviors, the employee score $f_{LOF} \left( {x_{i} } \right) = \left\{ {l_{1} ,l_{2} , \ldots ,l_{N} } \right\}$ is calculated. For a particular employee in different functional roles, the sample density of behavioral data is compared with the density of behavioral data of others in the same category to find out their abnormal behaviors.

$d\left( {x_{i} ,y_{i} } \right)$ denotes the Euclidean distance between two samples *x*_*i*_ and *y*_*i*_, and $d_{i} \left( {x_{i} ,y_{i} } \right)$ denotes the Euclidean distance from sample *x*_*i*_ to the *k*-th sample with the closest distance in its same functional role classification:

$$ d\left( {x_{i} ,y_{i} } \right) = \sqrt {\sum\limits_{k = 1}^{m} {\left( {x_{ik} - x_{jk} } \right)^{2} } } $$

$N_{k} \left( {x_{i} } \right)$ is the *k*-th neighborhood from *x*_*i*_, which is the set of all samples within the *k*-th distance from *x*_*i*_:

$$ N_{k} \left( {x_{i} } \right) = \left\{ {d\left( {x_{i} ,o^{\prime } } \right) \le d_{k} \left( {x_{i} } \right)} \right\} $$

The samples in the *k*-th neighborhood of *x*_*i*_ satisfies $\left| {N_{k} \left( {x_{i} } \right)} \right| \ge k$, and $\left| {N_{k} \left( {x_{i} } \right)} \right|$ is the number of *k* nearest samples. Given the parameter *k*, let the reachable distance $reach\_d_{k}$ be the one that has the larger value of $d\left( {x_{i} ,y_{i} } \right)$ and $d_{i} \left( {x_{i} } \right)$:

$$ reach\_d_{k} (x_{i} ,y_{i} ) = \max \left( {d_{i} \left( {x_{i} } \right),d\left( {x_{i} ,y_{i} } \right)} \right) $$

The local reachability density of *x*_*i*_ is $lrd\left( {x_{i} } \right)$. Assuming that $Y = \left\{ {y_{i} |i = 1,2, \ldots m} \right\}$ is a sample of the same roles in a neighborhood from *x*_*i*_, the average reachable distance *avg*(*Y*) of *Y* can be expressed as:

$$ avg(Y) = \frac{{\sum\limits_{{x_{j} \in N_{k} \left( {x_{i} } \right)}} { \, reach \, } \_d_{k} \left( {x_{i} ,x_{j} } \right)}}{{\left| {N_{k} \left( {x_{i} } \right)} \right|}} $$

$lrd\left( {x_{i} } \right)$ is inversely related to *avg*(*Y*), i.e., $lrd\left( {x_{i} } \right)$ is the inverse of *avg*(*Y*):

$$ lrd\left( {x_{i} } \right) = \frac{1}{avg(Y)} = \frac{{\left| {N_{k} \left( {x_{i} } \right)} \right|}}{{\sum\limits_{{x_{j} \in N_{k} \left( {x_{j} } \right)}} r each\_d_{k} \left( {x_{i} ,x_{j} } \right)}} $$

The outlier detection value can be expressed as the ratio of the local reachable density of the data sample to the neighboring samples of the same functional role:

$$ f_{LOF \, } \left( {x_{i} } \right) = \frac{{\sum\limits_{{x_{j} \in N_{k} \left( {x_{i} } \right)}} {lrd} \left( {x_{j} } \right)}}{{\left| {N_{k} \left( {x_{i} } \right)} \right| \cdot lrd\left( {x_{i} } \right)}} $$

If $f_{LOF \, } \left( {x_{i} } \right)$ is close to 1, it means that the density of *x*_*i*_ is similar to the density of the samples in its *k*-distance neighborhood, and *x*_*i*_ can be classified into the same cluster with the sample points in its *k*-distance neighborhood. If $f_{LOF \, } \left( {x_{i} } \right)$ is less than 1, it means that *x*_*i*_ is a dense point. If $f_{LOF \, } \left( {x_{i} } \right)$ is much larger than 1, *x*_*i*_ can be determined as an anomalous sample point.

The use of IF maximizes the variance, i.e., minimizes the similarity^27,28^. Given a dataset containing *n* samples, the degree of isolation of the delineated data points is rated by the path length of the tree. The smaller the value of the delineation path length of a data point, the larger the value of its outlier measure. IF measure whether a record *x* is an outlier by introducing an outlier function *s*(*x*, *n*):

$$ s(x,n) = 2^{{ - \frac{E(h(x))}{{c(n)}}}} $$

$$ c(n) = \left\{ {\begin{array}{*{20}l} {2H(n - 1) - 2(n - 1)/n,} \hfill & {n > 2} \hfill \\ {1,} \hfill & {n = 2} \hfill \\ {0,} \hfill & {other} \hfill \\ \end{array} } \right. $$

$$ H(x) = \ln (x) + \xi $$

where $E(h(x))$ is the expected value of the path length of sample point *x* in a multiple tree; *c*(*n*) is the average path length of the tree given the number of samples *n*; and *ζ* is Euler's constant, which is approximately 0.5772156649. A sample is normal when *s*(*x*, *n*) < 0.5, and is more likely to be abnormal as *s*(*x*, *n*) gets closer to 1. Using this approach, *s*(*x*, *n*) is calculated for the employee's behavior at each point in time to obtain the point in time when the abnormal behavior occurs^29,30^.

### Testing and analysis

The data used in this study comes from a new energy automobile company. The total number of employees in this company is 1200, which can be divided into three categories of managers (169, 14.08%), technicians (944, 78.67%), and operators (87, 7.25%) in terms of job classification. From the 1200 employees, 372 employees were selected according to random sampling method, of which the number of managers was 54 (14.52%); the number of technicians was 287 (77.15%); and the number of operators was 31 (8.33%). The results of the random sample generally correspond to the proportional division of the 1200 employees into the three categories of job classification. The time frame for data collection was May 1, 2021 to December 31, 2022 a total of 609 days. Data fields include: name, IP address, behavior object, behavior time, and behavior type, etc. The data was collected using Zabbix's process monitoring method at a frequency of 5 min. If the software is used, the software monitoring data value is 1. If it is not used, the data collection value is 0. The data is cleaned and filtered to finally get the utilizable data.

#### Detection of abnormal employees

**Figure 6:**
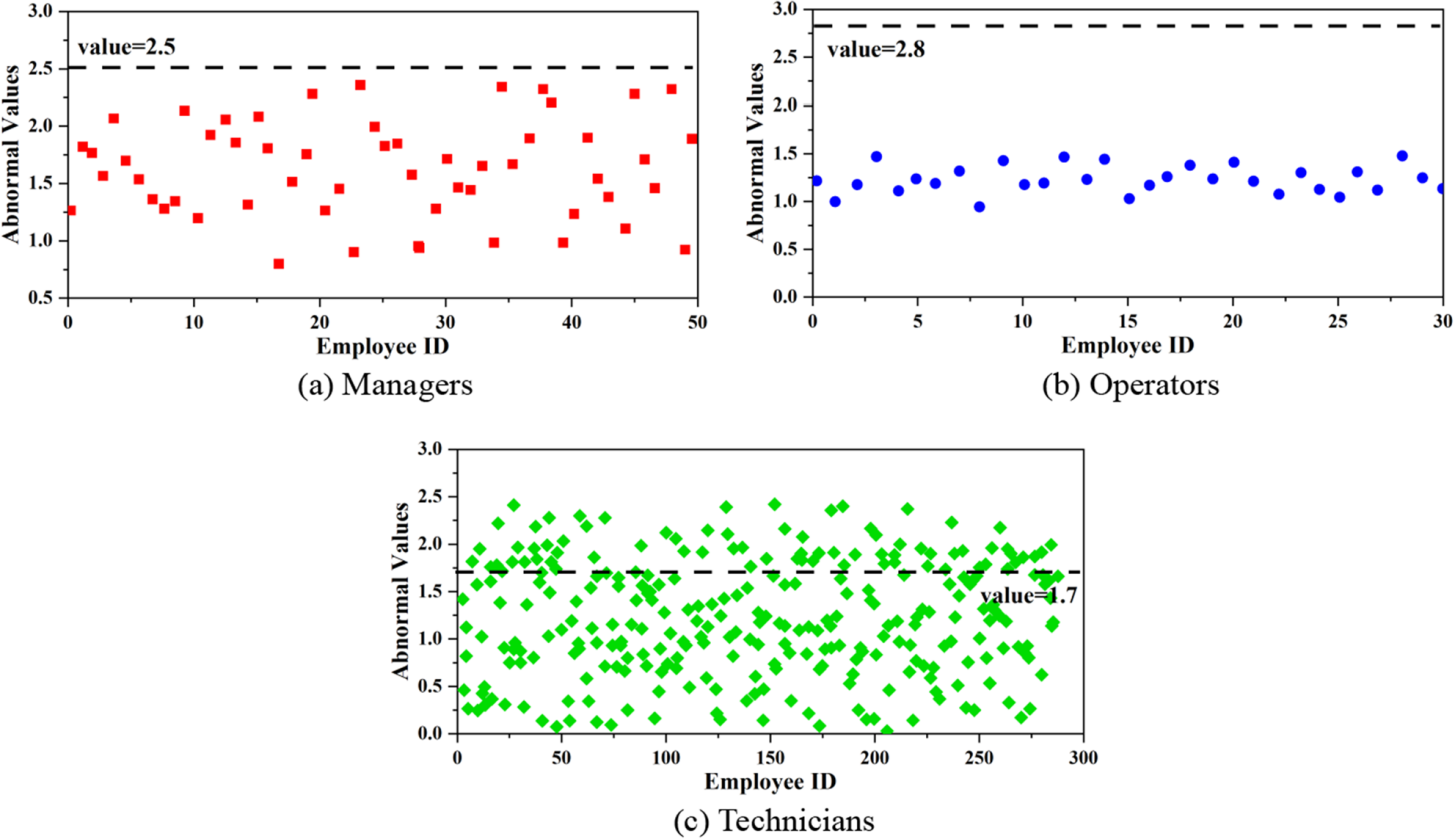
Distribution of abnormal employee behaviors. (**a**) Managers, (**b**) Operators, (**c**) Technicians.

Abnormal behaviors is shown in Fig. 6a–c are the abnormal detection results for managers, technicians, and operators, respectively. It can be seen that under the abnormal threshold, the managers and operators have no abnormal behaviors; the abnormal group of technicians is larger, indicating that there are more abnormal behaviors among such employees. For the analysis of employees with higher abnormal values, their main abnormal behaviors occurred on usage time of system and software, indicating that the usage time and frequency of technical software are much higher than others. Combined with the results of diligence analysis, it can be interpreted that such employees have larger workload and more positive work attitude.

#### Mining of abnormal behavioral patterns

Intercepting the employee's behavioral data from May 1, 2021 to December 31, 2022 for a total of 609 days as the analysis data set, dividing it by hours to analyze the abnormal characteristics of temporal behavior. Within this 609-day timeframe, it is divided by days to analyze the abnormal characteristics of the abnormal employees' temporal behavioral anomalies in the unit time interval per month, and no abnormal dataset is found from the time perspective after the anomaly detection by using IF.

**Figure 7:**
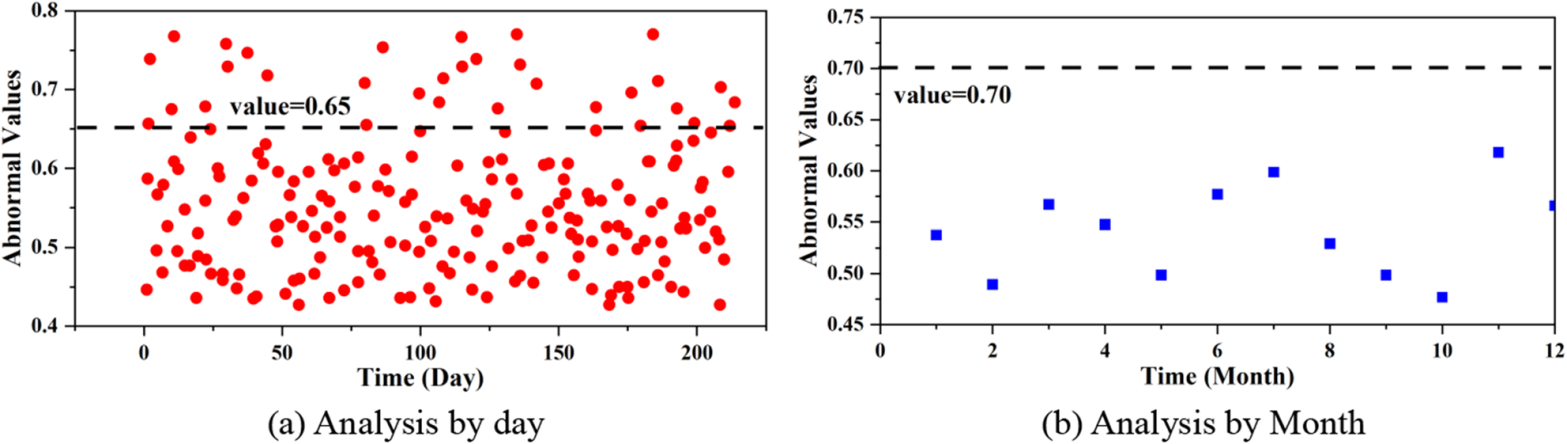
Temporal distribution of abnormal behaviors. (**a**) analysis by day, (**b**) analysis by month.

Figure 7a and b are the results of abnormal behavior analyzed by day and by month, respectively, where the threshold values are 0.65 and 0.70. Combined with the results of experimental data, the behavioral information at different granularity times can be interpreted as follows:

1. Employees with abnormal behaviors have different data patterns embodied in different time granularity.
2. Analyzed on a small time scale, employee behaviors usually occur in spare time, such as weekends, after work, and so on. But enlarge the time scale, in the larger time scale, abnormal employees have a certain regularity of their behavior, that is, the behavioral pattern is reflected in the coarse-grained time.
3. The abnormal results on the fine-grained time and the non-anomalous results on the coarse-grained time line may be related to the frequency and volume of tasks that such employees are involved in. As a result, there is a high rate of overtime work during the conduct of business, especially in the technical operations. However, on a larger scale, this "abnormal behaviors" tends to become "non-abnormal behaviors", which may be related to the nature of the work of such employees, who are required to work overtime on a regular basis.

## Temporal behavior prediction

### Temporal feature extraction

$U = \left\{ {u_{i} |i = 1,2, \ldots ,n} \right\}$ is defined as the set of employee software usage attributes^31^, where *u*_*i*_ denotes the *i*-th software usage attribute:

$$ u_{i} = \{ D = Day,b = Begin,e = End\} $$

*Day*: the day when the behavior of software usage occurs. *Begin*: time to begin using the software, such as *b* = 8:00 indicates that an employee starts using a software at 8:00. *End*: time to end use of the software, such as *e* = 10:00 that an employee end use of a software time for 10:00.

Employee *P* uses the software for *m* times:

$$ S = \left\{ {s_{j} |j = 1,2, \ldots ,m} \right\} $$

where $s_{j}$ denotes the *j*-th software usage behavior of employee *P* recorded in the database.

Given a sequence $S = \left\{ {s_{j} |j = 1,2, \ldots ,m} \right\}$ of software usage by *P*, predict *j* + 1-st usage behavior $s_{j + 1}$.

**Figure 8:**
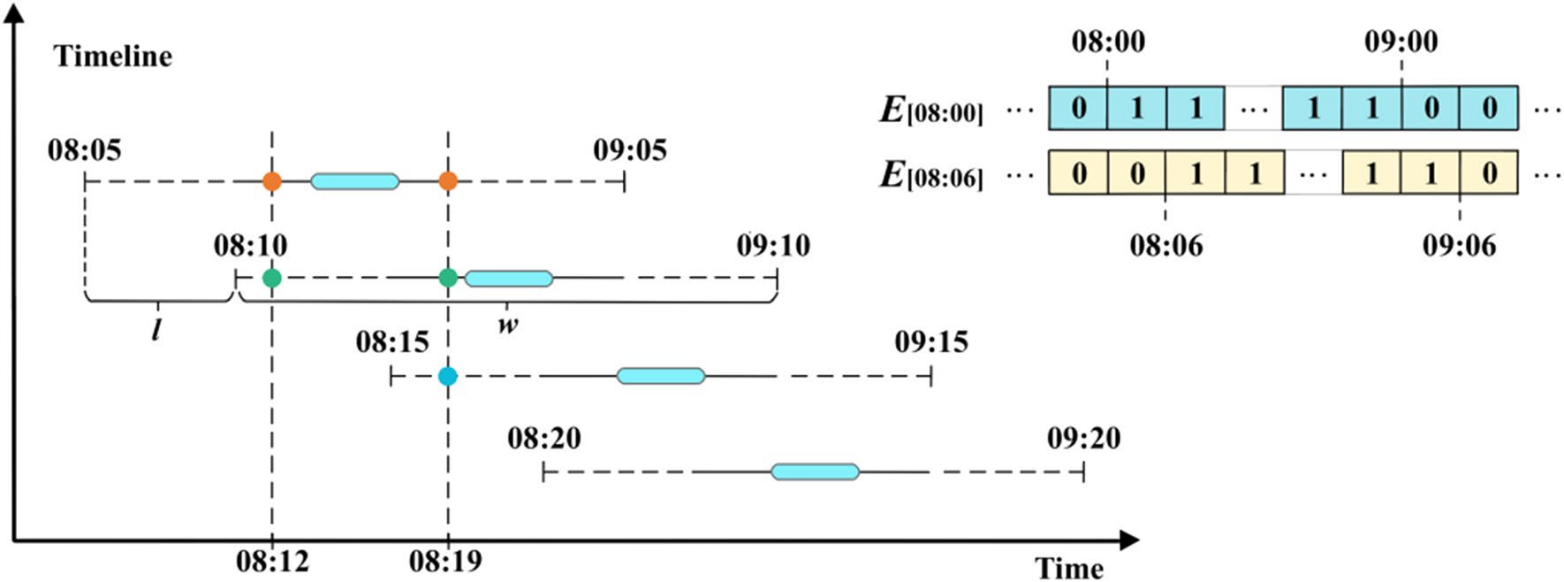
Overlapping coding.

Conventional feature extraction is performed for time intervals in units of 1 h. Due to the large span of the time, it makes the different behavioral features in the unit time to be masked, which ultimately leads to the extracted features with large errors. For example, both 08:00 and 08:48 are located in the [8:00, 8:59], which leads to the masking of the time difference^32,33^. This paper adopts overlapping time coding to solve the problem of feature error for larger time spans. The schematic diagram of overlapping time coding is shown in Fig. 8.

Assuming that the width of the sliding window is *w*, and that the time series is sampled in steps of *l* from 00:00 onwards, with a total sampling time of 24 + *w* hours, 24/*l* discrete time intervals with overlap can be obtained, denoted as $\left\{ {T_{1} ,T_{2} , \ldots ,T_{24/l} } \right\}$. Assume that a certain moment is *t* and its time vector is:

$$ e_{i} = \left\{ {\begin{array}{*{20}l} {1,t \in T_{i} } \hfill \\ {0, \, other} \hfill \\ \end{array} } \right. $$

where *e*_*i*_ is the *i*-th element of the vector *E*, i.e., $E = \left\{ {e_{i} |i = 1,2, \ldots } \right\}$, and *T*_*i*_ is the *i*-th discrete time slice. After reformatting the temporal attributes of the temporal behavior using overlapping coding, it can be turned into a non-sparse vector $v_{{u_{i} }}$, and then feature extraction is performed on $v_{{u_{i} }}$ to form a vector groups: $v_{{a_{1} }} ,v_{{a_{2} }} , \ldots v_{{a_{n} }}$.

### Prediction of behavioral patterns

LSTM has a great advantage in time-series data prediction, in order to improve the prediction accuracy, the LSTM model based on attention mechanism (Att-LSTM) is used to predict the employee's software use behavior^34^. The correlation between the historical behavior $s_{1} ,s_{2} , \ldots s_{m - 1}$ and the last behavior *s*_*m*_ is calculated using the attention mechanism, and the vector $\overrightarrow {{M_{n} }}$ of the current moment is constructed to represent the relationship between the current software usage behavior and the historical software usage behavior:

$$ \overrightarrow {{M_{n} }} = \sum\limits_{i = 1}^{n - 1} {\alpha_{i} } h_{i} $$

$$ \alpha_{i} = softmax\left( {h_{n} W_{c} h_{i} } \right) $$

where *h*_*n*_ is the output of hidden layer at the current moment; *h*_*i*_ is the output of hidden layer at moment *i*; *W*_*c*_ denotes the linear layer; and *α*_*i*_ is the weight, corresponding to the software usage data at moment *i*. Using the $\overrightarrow {{M_{n} }}$ and *h*_*n*_ for splicing, the result is input into the linear layer, and the output is calculated by the activation function, which results in the probabilistic output, where *W*_*o*_ denotes the output linear layer, $u_{n} = \overrightarrow {{M_{n} }} \oplus h_{n}$.

$$ o_{n} = softmax\left( {W_{o} u_{n} } \right) $$

The Att-LSTM model construction process is as follows^35^. The pseudocode form of the Att-LSTM algorithm is shown in Table 2A, and "/**/" indicates the annotation.

Step 1: Feature extraction and dataset division. The software uses behavioral features were extracted utilizing overlapping coding approach and the dataset was divided into training and testing sets according to 8:2.

Step 2: Data elimination. The discrete temporal behavioral feature data extracted using LSTM's oblivious gate filtering is shown in the following equation. *v*_*t*_ denotes the software-used feature vector of the input model at moment *t*, *W* denotes the weight matrix, and *b* denotes the bias vector.

$$ f_{t} = \sigma \left( {W_{if} v_{t} + b_{if} + W_{hf} h_{t - 1} + b_{hf} } \right) $$

Step 3: Data selection. The input gate of LSTM is utilized to save the processing feature data, which is stored in the neuron state as shown in the following equation, where *σ* is the sigmoid activation function.

$$ j_{t} = \sigma \left( {W_{ij} v_{t} + b_{ij} + W_{hj} h_{t - 1} + b_{hj} } \right) $$

$$ g_{t} = \tanh \left( {W_{ig} v_{t} + b_{ig} + W_{hg} h_{t - 1} + b_{hg} } \right) $$

where the neuron state *c*_*t*_ can be represented as:

$$ c_{t} = f_{l} *c_{t - 1} + j_{t} *g_{t} $$

Step 4: Output. the output gate of LSTM controls the output of the neuron:

$$ o_{t} = \sigma \left( {W_{io} v_{t} + b_{io} + W_{ho} h_{t - 1} + b_{ho} } \right) $$

where, *h*_*t*_ is the hidden state as shown in the following equation, where $\otimes$ is the element multiplication.

$$ h_{t} = o_{l} \otimes \tanh \left( {c_{t} } \right) $$

Step 5: Attention computation. The output vector $\overrightarrow {{M_{n} }}$ is computed using the attention mechanism.

Step 6: Prediction output. Based on the obtained self-attention matrix, the probability output *o*_*n*_ is calculated.

Step 7: Loss value is calculated. The loss function is:

$$ \xi = crossentorpy\left( {o_{n} , \, tar \, } \right) $$

where *crossentropy* denotes the cross entropy function and *tar* denotes the unique heat encoding value corresponding to the actual attribute value.

Step 8: Model training.

Step 9: Behavior prediction.

### Testing and analysis

The behavior data of 372 technicians of a new energy automobile company is used to verify the prediction model, and the time period is chosen from May 1, 2021 to December 31, 2022. Based on the dataset to construct the usage behavior sequence, for each technician, a sliding window with a width of 40 is used for sampling, and the sample sequence obtained is divided into a training set and a test set according to the ratio of 8:2.

**Figure 9:**
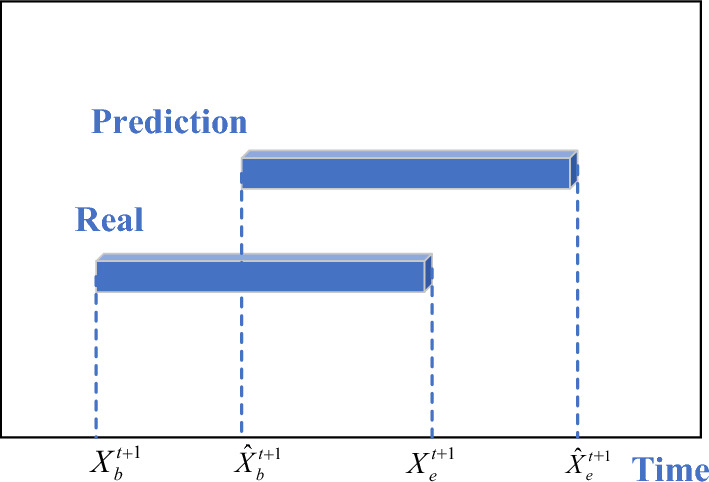
Truth and prediction of software usage behaviors.

The RMSE, MAE and MAPE are used to analyze the error between the prediction and the truth of the model, and the APA is used to make statistics on the error. Where $\hat{X}^{t + 1}$ is the prediction at the moment *t* + 1, $X^{t + 1}$ is the truth at the moment *t* + 1 and *n* is the number of samples. Further, to make the error calculation more suitable $\hat{X}_{b}^{t + 1}$, $\hat{X}_{e}^{t + 1}$, $X_{b}^{t + 1}$, and $X_{e}^{t + 1}$ are utilized to represent the "Prediction of begin time", "Prediction of end time", "Truth of begin time", and "Truth of end time", respectively, is shown in Fig. 9.

The improved equation is shown below:

$$ RMSE^{\prime } = \sqrt {\frac{1}{n}\sum\limits_{i = 1}^{n} {\frac{{\left( {\hat{X}_{b}^{t + 1} - X_{b}^{t + 1} } \right)^{2} + \left( {\hat{X}_{e}^{t + 1} - X_{e}^{t + 1} } \right)^{2} }}{2}} } $$

$$ MAE^{\prime } = \frac{1}{n}\sum\limits_{i = 1}^{n} {\frac{{\left| {\hat{X}_{b}^{t + 1} - X_{b}^{t + 1} } \right| + \left| {\hat{X}_{e}^{t + 1} - X_{e}^{t + 1} } \right|}}{2}} $$

$$ MAPE^{\prime } = \frac{1}{n}\sum\limits_{i = 1}^{n} {\frac{{\left| {\hat{X}_{b}^{t + 1} - X_{b}^{t + 1} } \right| + \left| {\hat{X}_{e}^{t + 1} - X_{e}^{t + 1} } \right|}}{{X_{b}^{t + 1} + X_{e}^{t + 1} }}} $$

The partial evaluation results are shown in Table 4:

**Table 4:** Comparison of model evaluation (part).

No.	Indicators	Model		
		SA^21^	LSTM^36^	Att-LSTM
175,841	MAE'	0.9132	0.8746	0.8012
	MAPE'	0.2583	0.1472	0.0683
	RMSE'	0.9747	0.9043	0.8377
175,842	MAE'	0.9463	0.8659	0.8104
	MAPE'	0.2491	0.1568	0.0759
	RMSE'	0.9766	0.9104	0.8246
…	…	…	…	…
176,583	MAE'	0.9072	0.8465	0.7981
	MAPE'	0.1764	0.1597	0.0627
	RMSE'	0.9817	0.9103	0.8272

**Figure 10:**
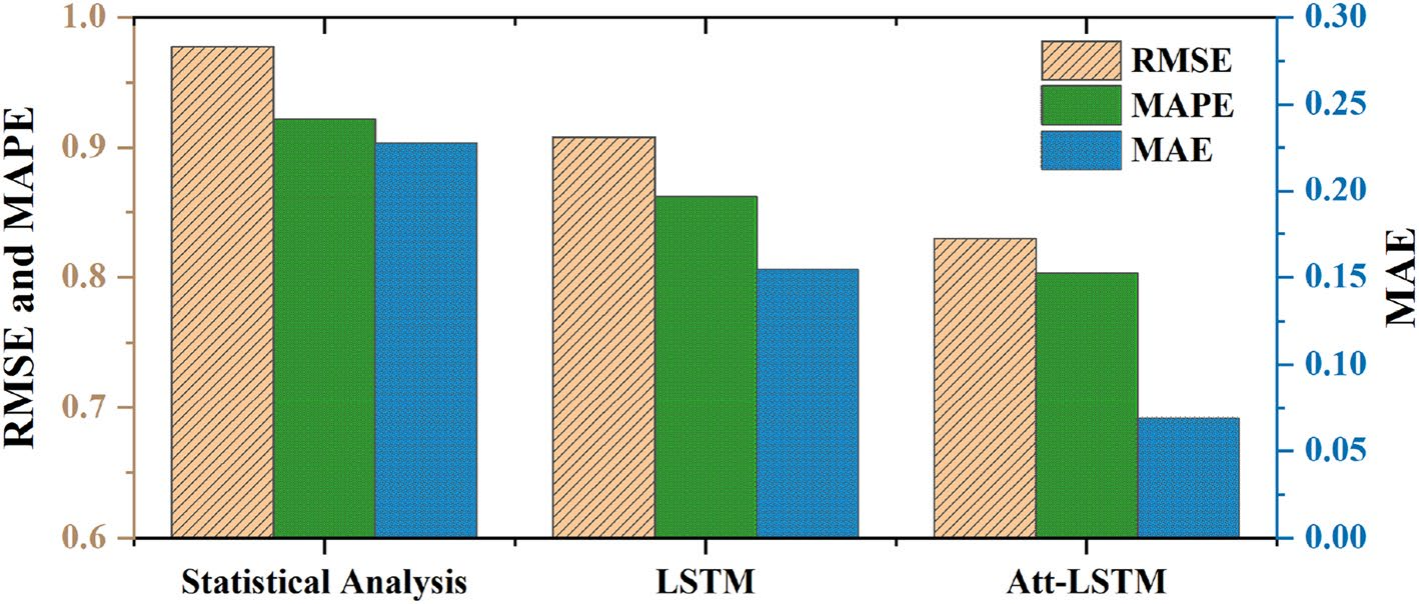
Evaluation comparison of different methods.

The APA is utilized to do statistics for the three indicators, and the results are shown in Fig. 10. Analyzing Fig. 10, it can be seen that Att-LSTM has the best prediction effect, the prediction effect of the SA is relatively poor, and the prediction effect of LSTM is better than that of the SA, but not as good as that of Att-LSTM. Att-LSTM achieved relatively ideal results in the prediction of software use behavior, but because human behavior has a great deal of ambiguity.

Behavioral variability is high in the role of technicians. Combined with the research analysis, the "abnormal" behavior of this type of roles is related to the work tasks and workload, and the uncertainty of the tasks leads to the uncertainty of the behaviors, which makes the abnormal people more abnormal when detecting the abnormalities. "Abnormal" people have different detection results at different granularity timelines. In a small timeline, certain employees have abnormal behavior, such as working overtime during weekdays and weekends. However, expanding the time span and analyzing from the coarse-grained timeline, the behaviors has a certain regularity, and the detection result is "non-abnormal". This phenomenon can be explained by the fact that from the perspective of coarse-grained timeline, regular overtime work is also a regular behavior. This suggests that the work of this group is unique, and that the volume and nature of their tasks contribute to what appears to be "abnormal" work behavior.

While focusing on employee departures, it is also important to look at the flow of departures, i.e. what industries and companies they join after leaving. What are the strengths of these industries and companies that attract talent there. A more obvious comparison is that many companies have become the preferred destination of employees after leaving in recent years by virtue of their work-life balance, high wages, relaxed working atmosphere and lucrative stock returns. At the same time, these companies have a set of comprehensive human resources development system and employee training programs in human resources management, and pay great attention to employee training and employee care. Not only new energy automobile enterprises, but also enterprises in all sectors should learn more from advanced human resource management practices and develop appropriate human resource strategies accordingly, so as to ultimately reduce employee turnover, strengthen the talent base and maintain market competitiveness through the implementation of human resource policies.

## Conclusion

Aiming at the problems that exist in the construction of the employee portrait model of enterprises, such as the employee portrait is not comprehensive enough and the efficiency of data analysis is low, this paper adopts data mining, combines the deep learning, statistical analysis and other technologies to propose a data mining-based employee portrait portrayal model. The research results are as follows:

1. A portrait portrayal model for employees based on data mining is proposed. Combining data mining, deep learning, statistical analysis and other technologies, a portrait portrayal model is proposed, which provides an intelligent solution for enterprise-level employee behavior analysis as well as portrait portrayal.
2. A diligence analysis model (DAM) based on improved GAN is designed. Summarizing the characteristics of GAN, CGAN, DCGAN and VAE, a diligence analysis model (DAM) based on improved GAN, is designed to realize employee diligence. The diligence evaluation is delineated by combining Random Forest and Statistical Analysis, while the data label selection for DAM training is realized by combining AHP analysis. The results of diligence analysis of DAM have high accuracy (80.39%) and outperform SA (70.24%), K-means (51.79%) and GAN (67.25%). The Kappa coefficient of DAM reaches 0.7384, which is highly consistent and higher than SA (0.6075), K-means (0.3711) and GAN (0.5661).
3. A method of abnormal behavior identification and temporal behavior prediction is designed. Firstly, Random Forest is used to screen the behavioral characteristics of employees; the LOF is used to detect abnormal behaviors on the employees; Isolation Forest is used to analyze abnormal employees from the time perspective, so as to mine the abnormal behavior patterns; the abnormal and non-abnormal behavioral patterns at different granularity times are summarized; an LSTM model (Att-LSTM) based on the attention mechanism is used to complete the prediction of employees' software usage behaviors. Att-LSTM predicts the best with an RMSE of 0.82983, which is better than LSTM (0.90833) and SA (0.97767); AM-LSTM has a MAPE of 0.80323, which is better than LSTM (0.86233) and SA (0.92223).

## Research shortcomings and future prospects

In conducting this study, the scientific and accuracy of the research methodology and results have been ensured as much as possible, but the following points still exist:

### Sample size

Only the employees of new energy enterprises were selected as the samples. Although the new energy industry is growing rapidly and the number of employees is rising, there is still a need to analyze each industry in terms of obtaining the specific characteristics of the abnormal behavior and tendency to leave of employees in new energy enterprises and the general characteristics of each industry.

### Diversity of research indicators

Only the employee's behavior data is selected. There is a limitation of accuracy in using only the operation behavior of employees as the basis for judging abnormal behavior and tendency to leave the company. This is because some employees have the habit of repeating operations, which can easily increase the rate of misjudgment. Employee's speech published on various platforms should also be considered, and the combination of the two can improve the prediction accuracy.

In addition, it provides new perspectives for subsequent studies on the causes of separation:

1. New energy enterprises were selected as the research perspective to deeply analyze the causes of their employee behavior and workplace mobility, and there are still a large number of employees working in the traditional manufacturing industries. Therefore, in the future, this type of research can be expanded to other industries, and can be carried out to compare and analyze the differences in the causes of separation in the industry perspective.
2. In this paper, we only selected employees' behavior data and did not conduct sentiment mining on employees' text comments. When employees browse the website or platforms, whether they will make text comments and whether they are mixed with the content of the tendency to leave the company can also reflect the causes of employees leaving the company to a certain extent. Therefore, sentiment mining for textual comments and combining it with behavioral analysis also deserves further research and analysis in the future.

### Supplementary Information

Supplementary Tables.

## Data Availability

Data cannot be shared publicly because of the raw data involves basic information about the employees of the enterprises. Data are available from the Research Ethics Committee of Universiti Putra Malaysia (contact via pspk@upm.edu.my) for researchers who meet the criteria for access to confidential data.
