# Supplementary material for: Analysis of employee diligence and mining of behavioral patterns based on portrait portrayal
Source: Sci Rep. 2024 May 24;14:11942. doi: 10.1038/s41598-024-62239-0 (PMC11126661; doi:10.1038/s41598-024-62239-0)
Supplement: Supplementary file 1 — Supplementary Tables. [file 41598_2024_62239_MOESM1_ESM.docx]

APPENDIX

**Table A1 Pseudocode for Algorithm 1 (DAM)**

| Algorithm 1 DAM |
| --- |
| Input: |
| *X_t_*=[*x*_1_, *x*_2_ ,..., *x_m_*_-1_, *x_m_*]*^T^*: The original dataset after selection, i.e., the training sample labels. |
| Output: |
| *X_classification_*: Diligence classification result |
| Begin |
| 01 *def Vae*(*X*): /* Define VAE network model */ |
| 02 set X' = *encoder*(*X*); |
| 03 set Z; /*hidden variable*/ |
| 04 for *i* = 0 to X' do. |
| 05 *z_i_* = *μ*(*x_i_*’)+*ε*×*σ*(*x_i_*’); /*hidden variable element*/ |
| 06 return Z |
| 07 *def* *G*(*Z*, *X*): /*define generator model**/ |
| 08 set *G_data*; |
| 09 for *i* = 0 to X do: |
| 10 *g_data_i_* = *generate*(*z_i_*, *x_i_*); /**generate data and save/ |
| 11 end for |
| 12 return *G_data*; /* return generate result */ |
| 13 def *D*(*X*,[*X*']): /* define discriminator model */ |
| 14 set *D_data*; |
| 15 for *i* = 0 to *X* do. |
| 16 *d_data_i_* = *discriminate*(*x_i_*, *x_i_*'); /*discriminate data and save */ |
| 17 end for |
| 18 return *D_data*; /* return the result of discrimination */ |
| 19 *def Gan*([*Z*, *X_t_*]): /* model training, train generator and discriminator */ |
| 20 set *Z* = *vae*(*X_t_*); /*Generate hidden variables**/ |
| 21 set Result; |
| 22 while (!*min_G_max_D_V*(*D*,*G*)): /* train the model until a Nash equilibrium is reached**/ |
| 23 for *i* = 0 to *x_t_* do: |
| 24 set *G_data* = *G*(*Z*, *X_t_*); /*generator training*/ |
| 25 set *result_i_* = *D*(*X_t_*, [*G_data*]); /*discriminator training*/ |
| 26 end for |
| 27 end while |
| 28 return Result; |
| 29 return *Gan*([*Z*], *X_t_*); /* return final result*/ |
| End |

**Table A2 Pseudocode for Algorithm 2 (Att-LSTM)**

| Algorithm 2 Att-LSTM |
| --- |
| Input: |
| *S_t_*=[*s*_1_, *s*_2_ ,..., *s_m_*_-1_, *s_m_*]*^T^*: The original dataset after selection, i.e. training sample labels |
| Output: |
| *S_classification_*: Software behavior prediction results |
| Begin |
| 01 while *epoch < EPOCH* do: |
| 02 *S_v_* = *feature Extraction*(*s*); |
| 03 for *i* in *n* do: |
| 04 *h_i_* = *LSTM*(*s_i_*); |
| 05 end for |
| 06 *H*←[*h*_1_, *h*_2_ ,..., *h_n_*_-1_]; |
| 07 $\overrightarrow{M_{n}}=Attention\left(H,h_{n}\right)$; |
| 08 $u_{n}=\overrightarrow{M_{n}}\oplus h_{n}$ |
| 09 $o_{n}=soft\max\left(W_{out\text{ }}u_{n}\right)$ |
| 10 $\xi=scrossentropy\left(o_{n},tar\right)$ |
| 11 if $\xi<\xi*$: |
| 12 end while |
| 13 output *S_classification t_* |
| 14 else: |
| 15 $backgpropagation(\xi)$ |
| End |
